# Household financial burden among multidrug-resistant tuberculosis patients in Guizhou province, China: A cross-sectional study

**DOI:** 10.1097/MD.0000000000021023

**Published:** 2020-07-10

**Authors:** Yun Wang, Edward B. McNeil, Zhongfeng Huang, Ling Chen, Xiaolong Lu, Chengqiong Wang, Huijuan Chen, Virasakdi Chongsuvivatwong

**Affiliations:** a Department of Health Service Management, School of Medicine and Health Management, Guizhou Medical University, Guiyang, Guizhou, China; b Epidemiology Unit, Faculty of Medicine, Prince of Songkla University, Hat Yai, Songkhla, Thailand; c Department of Tuberculosis, Guiyang Public Health Clinical Center, Guiyang; d Department of Respiratory Medicine, Affiliated Hospital of Zunyi Medical University, Zunyi; e Department of Tuberculosis Prevention and Control, Guizhou Center for Disease Prevention and Control, Guiyang, Guizhou, China.

**Keywords:** catastrophic costs, finance, Guizhou, MDR-TB, total costs

## Abstract

Multidrug-resistant tuberculosis (MDR-TB) threatens global public health. Poor access to health care due to financial hardship contributes to further transmission of the disease. The study aimed to:

1. quantify the magnitude of financial burden among affected households by MDR-TB in the first year of treatment;
2. measure financial and social protection of local policies for MDR-TB by catastrophic payments;
3. determine associated factors of catastrophic payments in Guizhou, China.

A cross-sectional study was conducted in 2 hospitals designated for MDR-TB from January to August 2018. Data were collected by interviewing eligible MDR-TB outpatients and reviewing the medical records. The magnitude of financial burden was documented by total cost and distribution of cost components. Catastrophic payments were measured by 2 indicators: catastrophic health expenditure (CHE) and catastrophic total costs (CTC), both of which were estimated by incidence and intensity. Their associated factors were determined using logistic regression models.

Of 161 households affected by MDR-TB, the average total costs due to MDR-TB treatment in the first year was US$ 8266 and consisted of 72% direct medical costs, 5% direct non-medical costs and 23% indirect costs (income loss). Thirty seven percent of direct medical costs were covered by insurance. Overall, the incidence of CHE and CTC was 68.3% and 87.0%, respectively. Both incidence and intensity for the 2 defined catastrophic costs increased when a households income decreased. Five significant factors of catastrophic costs were low household income, absence of students in a family, hospital length of stay, male gender, and job/productivity loss.

Households with MDR-TB patients shouldered a high financial burden which was mainly driven by direct medical costs and income loss in Guizhou. Greater catastrophic payments were associated with hospital length of stay and socioeconomic status, especially had a dose-response relationship with households income. Our findings suggest that financial and social protection of local policies for MDR-TB should be improved by preparing a uniform and comprehensive insurance package to cover sufficiently direct medical costs, and introducing social pro-poor assistance policies for risk families to protect them from financial hardship.

## 1 Introduction

Multidrug-resistant tuberculosis (MDR-TB) is an infectious airborne disease, which is caused by *Mycobacterium tuberculosis* and resistant to at least isoniazid and rifampin.^[1]^ Treatment generally lasts for 2 years and incurs high costs.^[2]^ MDR-TB disproportionately affects poor people^[3]^ and pushes them into a catastrophic poverty-disease loop.^[3]^ As a result, patients are unwilling to start or complete treatment due to financial hardship.^[4]^ Globally, low enrollment and treatment success rates are challenging problems that promote further drug-resistance, unnecessary transmission of MDR-TB, and high mortality.^[1]^ Therefore, it is important to quantify the financial burden of households due to MDR-TB in order to guide targeted policies toward mitigating these problems.

Cost data is used to document the magnitude of financial burden. A systematic review on financial burden due to TB showed that total costs incurred by treatment can be classified into 3 parts: direct medical costs (such as drug costs), direct non-medical costs (such as transportation to hospital), and indirect costs (such as income loss).^[5]^ However, many studies did not report the full distribution of these costs. In addition, total costs and the relevant components varied by country and region.^[5]^ In 2015, in a move to spur nationwide progress toward universal health coverage and the end TB strategy, the World Health Organization (WHO) launched a series of surveys investigating costs due to TB.^[3]^ Fourteen countries had completed the surveys by July 2019 of which 12 had reported their results, but not including China.^[6]^ Meanwhile, measurement of the costs for MDR-TB was insufficient.^[7]^ Thus, more research is needed.

Catastrophic health expenditure (CHE) has been used to measure the financial risk protection of a health policy under all conditions.^[3]^ CHE is defined as direct medical costs after reimbursement beyond a specified threshold of a households total expenditure, income,^[5,8–10]^ or capacity to pay^[5,10,11]^ in a given period, usually 1 year.^[12]^ After 2015, catastrophic total costs (CTC) was recommended by WHO as a TB-specific indicator for measuring financial risk and social protection for affected households.^[3]^ Thus, CTC highlights not only direct medical costs, but also direct non-medical costs and indirect costs which sum to 20% or more of a households income.^[3,4,13–16]^ To our knowledge, few studies have used CHE and CTC simultaneously. Understanding the incidence of 2 indicators and the factors associated with them could more effectively measure financial and social protection of local health policies.

China has the second highest MDR-TB burden in the world after India.^[1]^ The number of MDR-TB patients enrolled under treatment of the national TB program network was 5691 in 2015^[17]^ and the treatment success rate was only 41%.^[1]^ After the Global Fund ended their support for China in June 2014,^[18]^ health costs associated with MDR-TB treatment transited to co-payment by health insurance schemes and out-of-pocket (OOP) expenses. In addition, the beneficial level of insurance coverage depends on the local economic level.^[19]^ Guizhou is a low-income province with the third highest TB incidence in China.^[20]^ It is therefore an appropriate place to examine in depth how MDR-TB affects a households financial status.

The objectives of this study were to:

1. quantify the magnitude of households financial burden among MDR-TB patients by total costs and costs distribution during the first year of treatment;
2. measure financial and social protection of local policies by the incidence and intensity of CHE and CTC;
3. determine the associated factors of CHE and CTC in Guizhou, China.

## 2 Methods

### 2.1 Study design and setting

A cross-sectional study was conducted from January to August 2018. Participants were recruited from the only 2 prefecture level hospitals designated for MDR-TB diagnosis and treatment in Guizhou: Guiyang Public Health Clinical Center, and the Affiliated Hospital of Zunyi Medical University. A 24-month regimen was implemented in these 2 hospitals according to Chinas national guideline for MDR-TB treatment,^[21]^ consisting of a 6-month intensive phase followed by an 18-month continuation phase. One injectable drug is given during the intensive phase and at least 4 oral drugs are prescribed over the whole course of treatment. Outpatients visited the hospital every month to collect their drug supply.

The study protocol was approved by the Institutional Ethics Committee of the Faculty of Medicine, Prince Songkla University, Thailand (REC:60-338-18-1) and the Medical Ethics Committee, Guizhou Medical University.

### 2.2 Medical insurance

Table 1 summarizes the 3 basic medical insurance schemes in Guizhou:

1. the New Rural Cooperative Medical Scheme (NRCMS) for rural residents;
2. the Urban Employees Basic Medical Insurance (UEBMI) scheme for formal sector employees in urban areas; and
3. the Urban Residents Basic Medical Insurance (URBMI) scheme for urban residents excluding urban employees.^[22]^

**Table 1:**
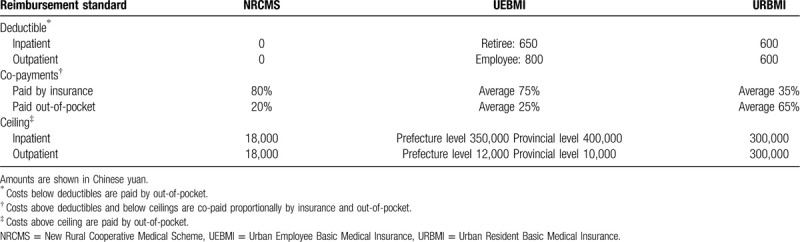
Reimbursement standards among 3 insurance systems in Guizhou province.

Deductibles, co-payments, and ceilings are different for the 3 insurance schemes. MDR-TB is covered by a case-based payments package in NRCMS with a ceiling of 18,000 yuan per year per patient with no deductible and is also included in specific outpatient reimbursement in UEBMI and URBMI schemes.

### 2.3 Participants, sample size, and recruitment

All MDR-TB patients registered from 2016 to 2017 in the 2 hospitals and who had finished their first year of treatment were eligible to participate in the study. The patients who had died or defaulted treatment before the study were excluded as they could not be traced.

The sample size of the study was calculated based on the single proportion formula where we assumed that the incidence of CTC would be approximately 83% based on results from previous studies.^[1,4,13–16]^ To estimate this proportion with a precision of 7% and 95% confidence would require 111 patients. We adjusted this estimate by assuming a design effect of 1.5 to account for the clustering of patients within hospitals. With these parameters, the final sample size was 166.

We identified 181 eligible MDR-TB patients, of which 20 (11%) refused to be interviewed. The remaining 161 patients (89%) were included in the analysis.

### 2.4 Variable definitions and measurements

#### 2.4.1 Total cost and relevant components

The notation (A, A_1_, A_1.1_, A_1.2_, A_2_ and A_3_) used here is also used in Table 3. Total costs due to MDR-TB (A) were defined as the costs borne by MDR-TB patients in their first year of treatment and included 3 parts: direct medical costs (A_1_), direct non-medical costs (A_2_), and indirect costs (A_3_) following the WHO protocol.^[3]^

**Table 3:**
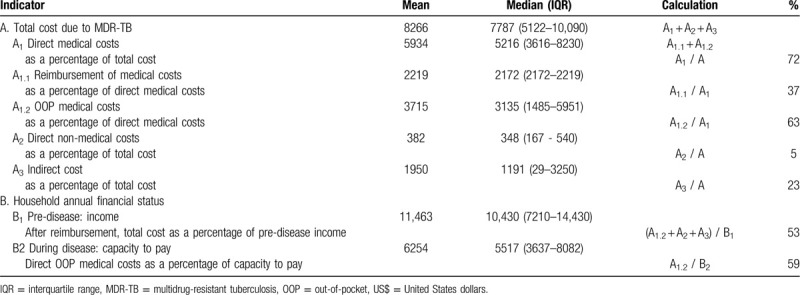
Total costs and costs distribution for MDR-TB treatment in the first year and household annual financial status (US$, n = 161).

Direct medical costs (A_1_) covered inpatient costs (bed charge, examination, medicines, material, laboratory tests and nurse charges), outpatient costs (registration, medicines and examination), injection fee and hepatic protector bought from a drug store. Direct medical costs were co-paid by partial reimbursement from an insurance scheme (A_1.1)_ and OOP expenses (A_1.2_).

Direct non-medical costs (A_2_) included transportation and accommodation costs incurred during hospital visits by the patients and/or their caregivers and were OOP expenses.

Indirect costs (A_3_) were defined as income lost due to MDR-TB causing the patients and/or their caregivers to be absent from work. The amount of income lost was calculated by multiplying the total daily wages of the patients and/or their caregivers by the number of workdays lost.

#### 2.4.2 Household annual financial status

The notation (B, B_1,_ and B_2_) used here is also used in Table 3. Household annual financial status (B) was measured by household annual income (B_1_) and capacity to pay (B_2_).

Household annual income (B_1_) is any kind of income earned by a household pre-diagnosis, such as fixed salary, the sale of goods or property, or a profit from investments.^[3]^ Household annual capacity to pay (B_2_) is defined as a household's non-food expenditure, which is equal to total household expenditure minus food expenditure.^[23]^ Total household expenditure includes food, daily necessities, transportation and communication, accommodation, education, OOP health expenditure and other spending.^[3]^

#### 2.4.3 Household catastrophic cost

If a households annual healthcare costs (direct OOP expenses and indirect costs) reached or exceeded 20% of the pre-disease annual household income then that household is defined as having incurred CTC.^[3]^ 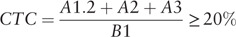


If a households OOP health payments (only direct medical OOP expenses) reaches or exceeds 40% of its capacity to pay then the household is defined as incurring CHE.^[23]^ 
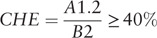


#### 2.4.4 Incidence and intensity of household catastrophic cost

Let T be the numerator, X be the denominator and Z be the threshold of the 2 catastrophic costs mentioned above. Then define Ei as an indicator for each household, i, which equals 1 if T_i_/X_i_ ≥ Z and 0 otherwise. The incidence of catastrophic cost was measured by the catastrophic payment head count (H), defined as the total number of households facing catastrophic cost divided by the total number of households (N) in the study.^[12]^ 
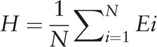


The intensity of catastrophic cost was measured by catastrophic payment overshoot (O) and mean positive overshoot (MPO) where O is the average degree by which T (as a proportion of X) exceeds Z.^[12]^ 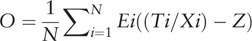


MPO is the excess expenditure per household experiencing catastrophic cost.^[12]^ 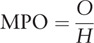


#### 2.4.5 The main independent variables

The main independent variables of interest comprised of 3 parts:

1. patients demographic characteristics: age, gender, marital status, ethnic, education level, occupation before and after diagnosis of MDR-TB and status of income after MDR-TB;
2. patients clinical characteristics: treatment regimen, length of stay in hospital, and number of outpatient visits;
3. household information: address, financial status, household size, household composition, and insurance status.

### 2.5 Data collection

Based on the inclusion criteria, eligible patients were approached and invited to participate. Those agreeing to participate were consecutively included and interviewed face-to-face using a structured questionnaire after providing written informed consent by a trained research team in the MDR-TB outpatient clinic of the study hospitals. The principal investigator checked the completeness of each questionnaire immediately after it was finished to ensure no missing data for variables used for analysis. In addition, the clinical information and direct medical costs were checked with medical records.

### 2.6 Statistical analysis

Data were entered into EpiData (version: 3.1, Odense, Denmark) and analyzed using R (version 3.4.2, Vienna, Austria). All costs were converted from Chinese yuan to US dollars using the average exchange rate of 6.63 yuan per 1 US dollar during the study period.

Descriptive statistics were presented using means, medians and interquartile ranges (IQR) as appropriate for continuous variables and frequencies and percentages otherwise. Incidence and intensity of household catastrophic cost were calculated based on the formulas shown above. Household and patient-level variables associated with the 2 previously defined catastrophic cost indicators (CTC and CHE) were explored first by univariate analysis. Variables with a *P* value below .2 from the univariate analysis were included in the initial multivariate logistic regression modeling process. The likelihood ratio test was assessed at each step and used to determine the final model where only variables with a *P* value less than .05 remained.

## 3 Results

### 3.1 Characteristics of patients

Demographic characteristics of the 161 patients are shown in Table 2. The median (IQR) age of participants was 36 (26–48) years and the male to female ratio was 2.2:1. The majority were married and had a low education level. Few patients had stable jobs and 55.3% became unemployed or could not work after being diagnosed with MDR-TB. Among those still employed, 18.2% had a decrease in their income. Amikacin, the only injectable drug, was used by all patients. Among the oral drugs received, prothionamide was the most common (93.8%), followed by levofloxacin (84.5%), pyrazinamide (82.0%), ethambutol (70.8%), and P-aminosalicylic acid (63.4%). Cycloserine, a costly drug, was used by one third of patients. The mean drug cost used in participants regimen in the first year was US$ 2812.3. The median (IQR) length of hospital stay was 18 (10–27) days. After discharge, patients visited the hospital for check-ups or to obtain drugs about once per month.

**Table 2:**
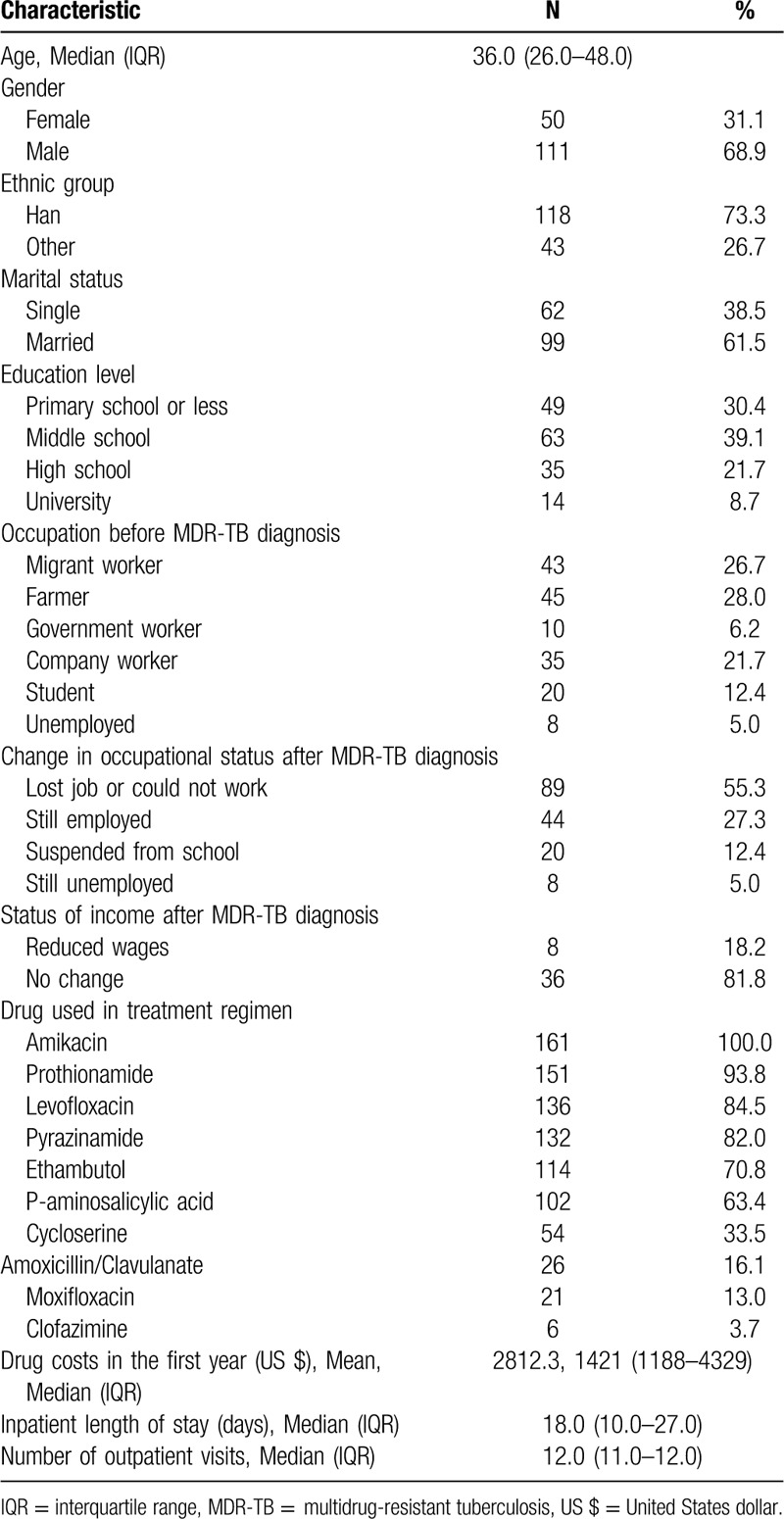
Characteristics of MDR-TB patients in Guizhou (n = 161).

### 3.2 Total costs, costs distribution, and annual financial status of households

Table 3 shows total costs and costs distribution among the 161 MDR-TB patients in the first year of treatment and their household annual financial status. The mean and median (IQR) of total cost were US$ 8266 and 7787 (5122–10,090), which consisted of 72% direct medical costs, 5% direct non-medical costs and 23% indirect costs. Direct medical costs were co-paid by reimbursement (37%) and OOP expenses (63%). After reimbursement, the average direct medical OOP expenses (US$ 3715) shared 45% of total costs. 53% of pre-disease annual income was spent when the participants fell ill and 59% of capacity to pay was spent on direct medical OOP costs during first year of MDR-TB treatment.

### 3.3 Incidence and intensity of catastrophic costs

Table 4 shows the incidence and intensity of the 2 catastrophic costs for MDR-TB care. Overall, the incidence (H) of CTC and CHE was 87.0% and 68.3%, respectively. The intensity for CTC and CHE, as measured by overshoot (O), was 45.3% and 27.7% and as measured by mean positive overshoot (MPO) was 52.1% and 40.5%, respectively. All 3 indicators (H, O, MPO) decreased when a households income increased. H, O and MPO for households in the poorest quartile (Q1) was around 1.5, 3.0, and 2.0 times higher than the respective indicators for households in the richest quartile (Q4).

**Table 4:**
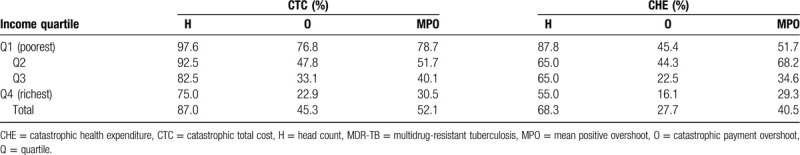
Incidence and intensity of catastrophic costs for MDR-TB care stratified by household annual income quartile.

### 3.4 Characteristics of households and their association with catastrophic costs

Table 5 presents the characteristics of households and univariate analysis with the 2 measures of catastrophic costs. Of the 161 study households, 77% were from rural areas. Two thirds of households had a moderate-sized family with at least 2 members earning an income. For more than half of households, the patients themselves were the primary income earners prior to being diagnosed with MDR-TB. 63% of families had at least 1 student and 97% of households were covered by medical insurance.

**Table 5:**
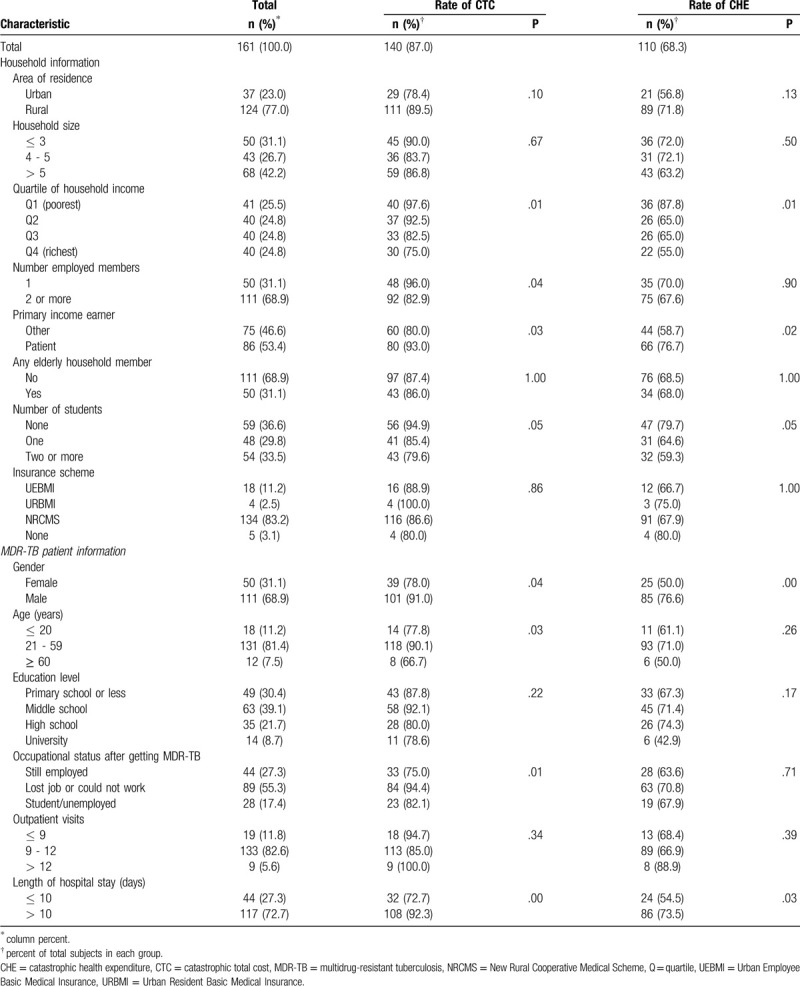
Characteristics of households and univariate analysis with catastrophic costs.

CTC and CHE were more common among the poorest families, those in which the MDR-TB patient was the family's primary income earner, families without students, families with patients who were male, and whose length of stay in hospital exceeded 10 days. CTC was also more common among families with only 1 member having a job, with patients who were aged 21 to 59 years, and those whose patients had lost their job due to their illness.

### 3.5 Determinants of catastrophic costs

Table 6 shows the results of the multivariate analysis investigating determinants of CTC and CHE. One factor, hospital length of stay exceeding 10 days, was statistically significant for both indicators. In addition, families with the lowest income (Q1), or the TB patient lost their job/could not work after being diagnosed with MDR-TB, were more likely to have CTC. Families without any students or male TB patients were significantly associated with CHE.

**Table 6:**
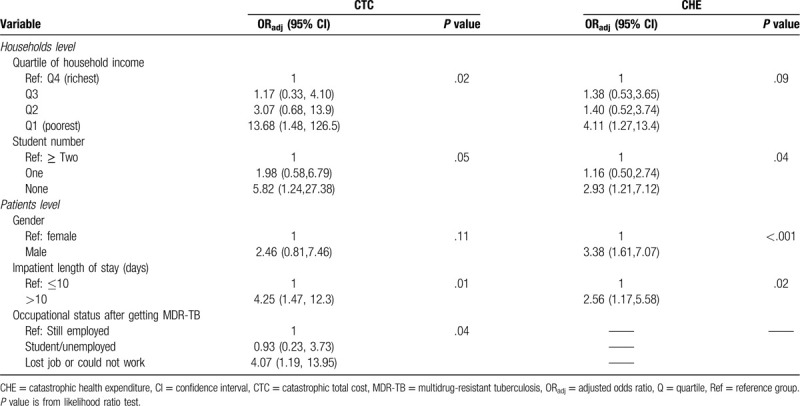
Multivariate logistic regression model predicting determinants of catastrophic costs for MDR-TB care.

## 4 Discussion

This study offers a view of the financial burden suffered by MDR-TB patients and their families in a TB endemic area of China. After reimbursement from an insurance scheme, the affected households bore half of the medical care expenses in addition to indirect costs, which was around one quarter of the total costs. More than two-thirds of the affected households experienced CHE and nearly 9 out of 10 faced CTC. Households had an increased risk for catastrophic costs if their family was poor, did not have students, if the patients had a hospital length of stay exceeding 10 days, were male, and had lost their job or could not work after being diagnosed with MDR-TB.

A systematic review of health services costs due to MDR-TB has shown that a positive relationship existed between cost and local economic status, and costs ranged from US$ 1218 in low-income countries to US$ 83,365 in high-income countries.^[24]^ Total costs in our study appear to be comparable to that in lower middle-income countries (US$ 6313).^[24]^ In terms of cost components, direct medical costs accounted for the highest proportion of total costs in our study, which is consistent with result from Viet Nam.^[25]^ Cost of drugs was an important driver of direct medical costs shown by a systematic review of financial burden for TB,^[5]^ which agrees with our finding. The proportion of indirect cost ranked the second of total costs in our study, which is similar to other countries such as Fiji, Ghana and Philippines.^[1]^ Direct non-medical costs constituted the smallest part of total costs in our study, which is similar with that in Myanmar and Mongolia.^[1]^

The incidence of CHE in our study was similar to that in Ethiopia (63%).^[11]^ The incidence of CTC was comparable to that in Indonesia (83%)^[16]^ and Kenya (86%),^[1]^ but lower than that in Viet Nam (98%).^[15]^ The high incidence of 2 indicators shows that financial and social protection of health policies in Guizhou are limited. Firstly, the beneficial level of insurance coverage for a household is insufficient. Low ceiling of outpatient reimbursements in UEBMI, low ratio paid by URBMI, and the limited maximum liability of NRCMS could not sufficiently cover the direct medical costs. Costly drugs for MDR-TB, such as cycloserine and moxifloxacin, are only partially covered by the insurance schemes.^[26,27]^ Secondly, more than half of patients in our study lost their jobs and productivity after being diagnosed with MDR-TB. Social protection could not effectively be a financial safety net for this poor group. Thus, income loss and high OOP direct payments for MDR-TB treatment pushed them into catastrophic payments.

In terms of determinants of catastrophic costs, our study firstly revealed that the incidence of catastrophic costs declined as income increased, regardless of the approach used. These results are consistent with many other studies.^[4,8,9,11,14]^ Secondly, the length of hospitalization exceeding 10 days significantly increased the likelihood of catastrophic cost, irrespective of the approach used. Under limited coverage from insurance, longer hospitalization equates to higher OOP expenses. Thirdly, the risk of CHE was higher for families without students. One of the possible reasons may be the fact that a family without students spent more on healthcare due to the absence of pressure to pay for a child's education. Another possible reason may be that a family without students had a lower annual expenditure, therefore the proportion of health payment shared out of capacity to pay was high. Fourthly, male MDR-TB patients contributed to high catastrophic payments in our study, which is consistent with a study by Kingsley et al^[10]^ who explained that men were the primary income earners in their families. Thus, loss of employment would subsequently send their household into catastrophe. It is interesting to note that a significant association was found in our study between patients losing their job after diagnosis and CTC. Income loss increases the risk of catastrophic costs.^[11,14,28]^

This study has some limitations. First, in line with the WHO protocol, our study subjects were those who were registered for MDR-TB treatment under national tuberculosis program networks. It has been reported elsewhere^[29]^ that only half of the patients diagnosed with MDR-TB were willing to receive treatment due to financial problems. Thus, the real situation may be worse than what we have reported in this paper. Second, the patients who had died or defaulted treatment before commencement of our study were not included as they could not be traced. Thus, the sample we obtained would be somewhat biased by survival and better ability to cope with the financial constraints. Third, recall bias is unavoidable for direct non-medical costs and indirect costs. Fourth, the rejection rate (11%) slightly exceeded our expectations (10%) so that the actual number of patients recruited was slightly less than the sample size calculated. Finally, our study was conducted in a low-income province, so the results may not represent the situation of middle or high-income provinces in China.

## 5 Conclusions

High financial burden was borne by households with MDR-TB patients in Guizhou mainly due to direct medical costs and income loss. Hospital length of stay and socioeconomic status were the main factors associated with catastrophic costs, and which had a dose-response relationship with household's income. Our findings suggest that financial and social protection of policies for MDR-TB in Guizhou should be improved, including the preparation of a uniform and comprehensive insurance package to sufficiently cover direct medical costs, and provision of pro-poor assistance policies, such as a targeted poverty alleviation program^[30]^ for high-risk families to protect them from financial hardship.

## Author contributions

**Conceptualization:** Virasakdi Chongsuvivatwong.

**Data curation:** Virasakdi Chongsuvivatwong.

**Formal analysis:** Edward B McNeil, Virasakdi Chongsuvivatwong.

**Funding acquisition:** Virasakdi Chongsuvivatwong.

**Investigation:** Feng Zhong Huang, Ling Chen, Long Xiao Lu, Qiong Cheng Wang, Huijuan Chen.

**Methodology:** Virasakdi Chongsuvivatwong.

**Project administration:** Virasakdi Chongsuvivatwong.

**Software:** Edward B McNeil.

**Supervision:** Huijuan Chen, Virasakdi Chongsuvivatwong.

**Writing – original draft:** Virasakdi Chongsuvivatwong.

**Writing – review & editing:** Edward B McNeil, Virasakdi Chongsuvivatwong.
